# Two successful pregnancies in a membranous nephropathy patient: Case report and literature review

**DOI:** 10.1097/MD.0000000000037111

**Published:** 2024-02-09

**Authors:** Congcong Qin, Zhijuan Hu, Yanan Shi, Hui Cui, Jiejie Li

**Affiliations:** a Department of Internal Medicine, Hebei North University, Zhangjiakou, China; b Department of Nephrology, Hebei General Hospital, Shijiazhuang, China; c Department of Internal Medicine, Hebei Medical University, Shijiazhuang, China; d Department of Internal Medicine, North China University of Science, Tangshan, China.

**Keywords:** antiphospholipase A2 receptor antibodies, kidney biopsy, membranous nephropathy, pregnancy, tacrolimus

## Abstract

**Background:**

Pregnancy in patients with nephrotic syndrome presents enormous challenges to both the mother and fetus, and there are no treatment guidelines for these patients.

**Methods:**

We show a case of a woman with anti-PLA2R antibody-positive membranous nephropathy who did not have a kidney biopsy. Her clinical course during both pregnancies was closely followed and her medications were guided.

**Results:**

She gave birth to 2 healthy babies and her condition was very well controlled with the help of medication.

**Conclusion:**

Patients with nephrotic syndrome can have successful pregnancies after drug treatment. In addition, similar to the non-pregnant population, percutaneous kidney biopsy is not required for the diagnosis of idiopathic membranous nephropathy (IMN) in pregnant nephrotic syndrome patients with anti-PLA2R antibody positive, but the etiology of secondary MN should be excluded.

## 1. Introduction

One of the main causes of nephrotic syndrome (NS) in adults is membranous nephropathy (MN), which primarily affects middle-aged patients. In recent years, MN has trend to occur in younger women, but is uncommon in women of childbearing age. There are few reports on pregnancy in women with MN, which is an extremely challenging problem. Although several therapeutic regimens can be chosen according to the risk stratification of MN, there are no treatment guidelines for MN with pregnancy. We present a case of a young woman with idiopathic MN (IMN) who had 2 successful pregnancies, aiming to provide some treatment options for this population.

## 2. Case report

A 30-year-old Asian woman was found to have urine protein at 6 weeks of gestation during her first pregnancy. She was not referred to a nephrologist until severe edema of both lower extremities was present at 19-week pregnancy. She was otherwise healthy, with normal findings on heart and lung examinations, but pitting edema (3+) in both legs. Laboratory testing showed a hemoglobin level of 86 g/L, 24-hour urine protein of 9.28 g/24 h, serum albumin of 12.9 g/L, serum creatinine of 0.51 mg/dL, and antiphospholipase A2 receptor (PLA2R) antibodies of 73.88 RU/mL (<20 RU/mL). Laboratory details at the time of admission are provided in Table 1. Cancer screening, antinuclear antibody, hepatitis serologic findings, and complement levels were within the normal ranges. We considered this patient with IMN.

**Table 1:** Laboratory findings on admissions.

Laboratory examination	Results
WBC (3.5-9.5 × 109/L)	5.46
Hb (115–150 g/L)	86
MCV (82–100fL)	91.2
MCHC (316–354g/L)	347
Plt (125–350 × 109/L)	197
CRP (0–10 mg/L)	5.23
ESR (0–15 mm/h)	106
Total protein (65–85 g/L)	40.6
Serum albumin (40–55 g/L)	12.9
Globulin (20–40 g/L)	27.7
BUN (8.68–24.64 mmol/L)	2.5
Creatinine (0.6~1.1mg/dL)	0.530
eGFR, mL/(min·1.73m^2^)	127.75
Uric acid (155–357 μmol/L)	212.8
Fasting blood glucose (3.9–6.1 mmol/L)	4.06
Total cholesterol (3.0–5.7 mmol/L)	7.44
Triglyceride (0.1–1.7 mmol/L)	3.45
24h urine protein (0–0.15 g/24h)	9.28
Tumor markers	negative
ANA	<1:100
Serum immunofixation electrophoresis	negative
dsDNA	negative
PT (9.8–12.1 s)	8.7
INR (0.85–1.3)	0.76
APTT (23.3–32.5 s)	23.6
FIB (2–4 g/L)	5.16
TT (14–21 s)	14.3
DDi (0–0.55 mg/L)	1.62
HBsAg (<0.05 IU/mL)	0.01
HBsAb (<10 mIU/mL)	21.79
HBeAg (<1COI)	0.28
HBeAb (>1COI)	1.61
HBcAb (<1COI)	0.12
HCV	0.04
HIV	0.07

ANA = antinuclear antibody, APTT = activated partial thromboplastin time, BUN = blood urea nitrogen, CRP = C-reactive protein, DDi = D-Dimer, dsDNA = double-stranded DNA, eGFR = estimated glomerular filtration rate, ESR = erythrocyte sedimentation rate, FIB = fibrinogen, Hb = hemoglobin, HBV = hepatitis B virus, HCV = hepatitis C Virus, HIV = human immunodeficiency virus, INR = international normalized ratio, MCHC = mean corpuscular hemoglobin concentration, MCV = mean corpuscular volume, Plt = platelet count, PT = prothrombin time, TT = thrombin time, WBC = white blood cells

**Figure 1.**
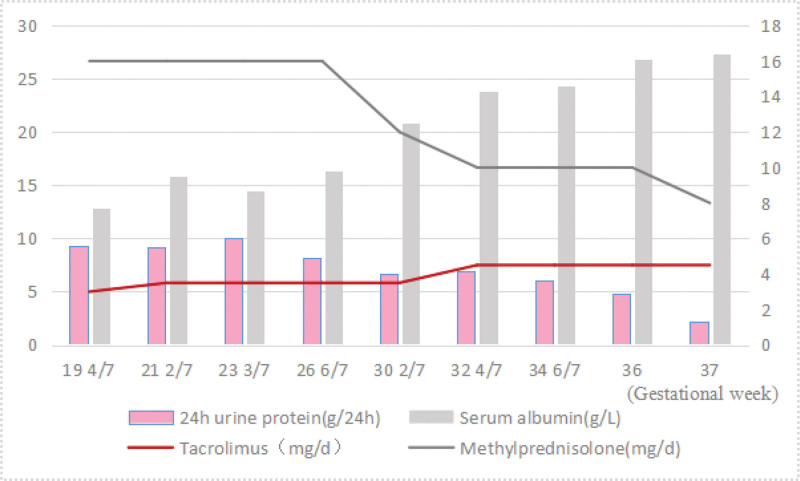
Laboratory data and medication adjustments from the patient during her first pregnancy.

Although kidney biopsy is not a contraindication for pregnancy,^[1]^ renal biopsy was not performed after weighing the relevant risks. The patient agreed to start immunosuppressive treatment with methylprednisolone 16 mg qd, and tacrolimus 1.5 mg q12h. Other medications included enoxaparin for preventing thrombosis and a polysaccharide-iron complex capsule for anemia. The patient was discharged with regular follow-up, the data of which is shown in Figure 1. The dose of tacrolimus was adjusted from 3 mg/d in the 19th week to 4.5 mg/d in the 32nd week of gestation, which was continued until delivery. The dose of methylprednisolone was gradually reduced from the initial 16 to 8 mg/d before delivery. Her urine protein decreased, serum albumin increased and leg edema improved. The last visit before delivery revealed hemoglobin 112 g/L, 24-hour urine protein 2.2 g/24 h, serum albumin 27.3 g/L, and serum creatinine 0.53 mg/dL.

At 38-week gestation, a 3100-g-weight boy was successfully delivered vaginally with Apgar scores of 9 and 10 at 1 and 5 minutes, respectively. The patient did not develop hypertension, hyperglycemia, and other complications throughout her pregnancy. The dose of tacrolimus was tapered after delivery and maintained at 0.5 mg/d 1 year later. Methylprednisolone was completely discontinued 8 months after delivery. The patient’s proteinuria was in long-term partial remission (Table 2).

**Table 2:** Proteinuria remission data and medication adjustments in patients after the first delivery.

Postnatal period (month)	Urine protein/24 h (g/24h)	Creatinine (mg/dL)	Serum albumin (g/L)	Tacrolimus (mg/d)	MP (mg/d)
0	2.2	0.535	27.3	4.5	8
0.5	1.17	0.666	29.8	4.5	8
1	1.15	0.524	32.9	4	6
2.5	1.1	NA	NA	4	4
6	0.23	0.589	42	2.5	4
8.5	0.75	NA	NA	2.5	Discontinuance
11	0.63	0.528	43.3	2.5	
12	0.95	0.475	42.9	2.0	
13.5	0.68	0.510	43.5	0.5	
14	0.36	0.521	45.2	0.5	

MP = Methylprednisolone, NA = not available

**Figure 2.**
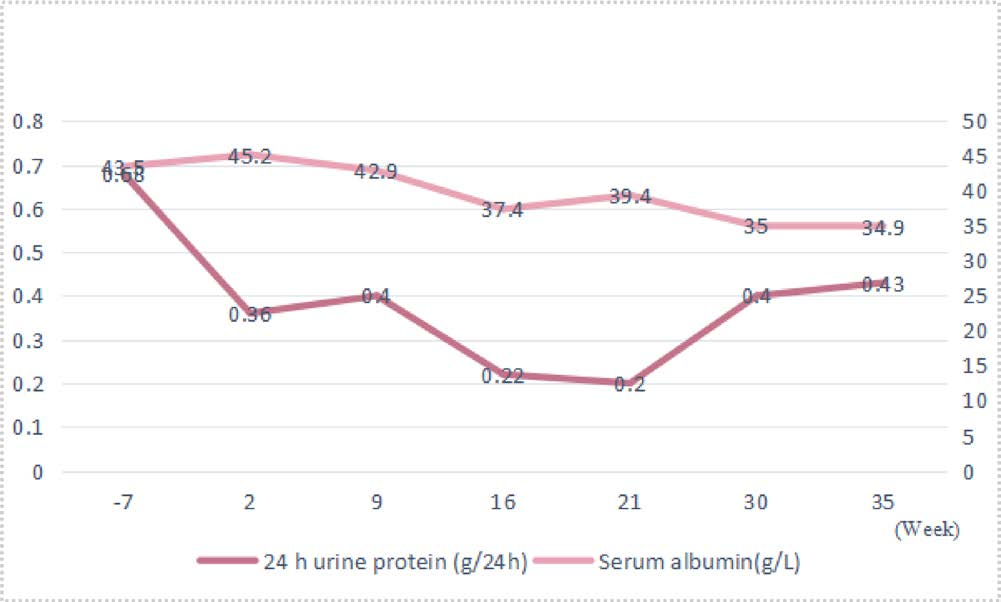
24 h urine protein and Serum albumin during the patient’s second pregnancy.

Accidentally, the patient was pregnant again after giving birth for 14 months. Tacrolimus 0.5 mg/d alone was administered during this pregnancy. The disease was well controlled with a urinary protein of 0.2 to 0.43 g/24 h and serum albumin of 34.9 to 45.2 g/L during the second pregnancy (Fig. 2). Her serum creatinine was between 0.495 and 0.521 mg/dL, and PLA2R was 3.24, <2.0, and <2.0 RU/mL at the 2nd, 15th, and 30th week of pregnancy, respectively. She gave birth to a healthy baby girl at 39 weeks of pregnancy. Until now, the patient is still on a tacrolimus 0.5 mg/d medication.

## 3. Discussion

The subepithelial deposition of immune complexes and the diffuse thickening of the basement membrane are the primary pathological characteristics of MN. Its clinical manifestations are massive proteinuria and hypoalbuminemia. According to the etiology, MN is divided into 2 categories–IMN and secondary MN (SMN)–of which, IMN is the most common type. Its pathogenesis is not fully understood and is generally considered to be related to autoimmunity.

The best instrument for determining MN is the percutaneous kidney biopsy. Studies on kidney biopsy during pregnancy are few and most of them are of small sample size. In a systematic review^[2]^ of 39 studies of 243 kidney biopsies in pregnancy, Piccoli et al pointed out that when compared to the postpartum period, pregnancy had a significantly higher risk of kidney biopsy (7% vs 1%; *P = *.001). All serious complications related to biopsy were recorded at 23 to 26 weeks of gestation. Therefore, renal biopsy at 23 to 26 weeks should be carefully considered. In addition, with the gradual enlargement of the uterus after 12 weeks of gestation, the prone position may cause fetal ischemia and hypoxia due to placental compression, so the lateral position or sitting position may be more suitable for patients, which may put higher demands on patients and biopsy doctors. In any case, the potential risks of renal biopsy during pregnancy are present. A previous study^[3]^ reported 15 renal biopsies during pregnancy, of which 4 had identifiable hematomas and 1 required blood transfusion. Therefore, we believe that reasonable control of blood pressure and good coagulation function is necessary before the renal biopsy.

To date, there are no uniform standards for blood pressure control during pregnancy in patients with renal disease. In 2019, the American College of Obstetricians and Gynecologists^[4]^ pointed out that blood pressure during pregnancy should be controlled below 160/110 mmHg and even lower in patients with concomitant diabetes and kidney disease. However, most international guidelines still recommend that blood pressure should be kept below 140/90 mmHg during pregnancy.^[5]^

Nifedipine, labetalol, and methyldopa are recognized as safe drugs during pregnancy.^[1]^ Nifedipine has more advantages in moderate to severe hypertension. Other β-blockers may have potential teratogenic risks. Angiotensin-converting enzyme inhibitors (ACEIs) and angiotensin II receptor blockers (ARBs) are contraindicated during pregnancy due to their teratogenic effects, especially in the cardiovascular and central nervous systems, and may increase the risk of stillbirth.^[6]^ Diuretics are generally used as second-line therapy for gestational hypertension,^[4]^ and to date, there is no definite evidence that they can increase the risk of pregnancy. However, spironolactone is contraindicated during pregnancy due to its potent antiandrogen effect.^[7]^

In 2009, the discovery of antibodies against PLA2R by Beck et al^[8]^ was a milestone for MN. Antibodies against PLA2R are positive in 70% to 80% of patients with IMN, while they are rarely detected in secondary MN.^[9–11]^ The 2021 KDIGO practice guidelines^[12]^ state that it is not necessary to perform a renal biopsy to confirm the diagnosis of MN in patients who have NS and whose antiPLA2R antibodies are positive, but is still necessary for patients requiring immunosuppressive therapy. In our patient, the kidney biopsy was not performed because of her older gestational age at presentation and her lack of willingness for renal biopsy. Fortunately, her NS was well controlled after drug treatment without a kidney biopsy. It shows that similar to the nonpregnant population, for pregnant patients with NS who are positive for antiPLA2R antibody, percutaneous renal biopsy may not be done when diagnosing IMN.

During 2 pregnancies of our patient, different doses of tacrolimus were continuously used to reduce the urinary protein of the patient and achieved good results. Tacrolimus is a calcineurin inhibitor (CNI) that can cross the placenta to the fetus.^[13]^ So far, there is no conclusive evidence that tacrolimus has a teratogenic risk.^[14]^ Tacrolimus has reportedly been used successfully and safely to treat IMN during pregnancy.^[15]^ In our patient’s first pregnancy, tacrolimus and corticosteroids were combined because the patient had a large amount of urinary protein and a higher titer of antiPLA2R antibody (73.88 RU/mL). During the second pregnancy, the patient’s condition was relatively stable, and we only used very low-dose tacrolimus monotherapy for maintenance therapy (0.5 mg/d). The most common maternal–fetal adverse reactions to tacrolimus include potential nephrotoxicity, premature delivery, neonatal hyperkalemia, etc.^[16,17]^ The aforementioned risks were not shown in our report, which may be related to our lower dose of tacrolimus. Additionally, cyclophosphamide is a classic choice for MN with a strong immunosuppressive effect. However, it is contraindicated during pregnancy (FDA category D) and should be discontinued before a planned pregnancy at least 3 months due to teratogenic effects.^[18]^ There are limited data on the use of rituximab for the treatment of MN in pregnancy and no evidence of a teratogenic risk.

Unlike other case reports, our patient had experienced 2 pregnancies and had very low serum albumin at baseline. Admittedly, she is great lucky, since she has good sensitivity to tacrolimus. This suggests that tacrolimus has potential value in the management of IMN in pregnancy, even though there have been reports of a risk of relapse after tacrolimus is discontinued.^[19]^ There is no doubt that pregnancy in women with NS increases maternal–fetal risk, but it does not mean that pregnancy is prohibited. Our report suggests that for women with a strong desire to have children, the disease activities can be controlled by drugs to improve pregnancy outcomes and lower the likelihood of complications.

## 4. Conclusion

For antiPLA2R-positive pregnant women with typical NS, kidney biopsy is not necessary for the diagnosis of IMN, but the etiology of secondary MN should be excluded. The combination of glucocorticoid and tacrolimus may as a treatment option for MN during pregnancy. MN patients with NS can have a successful pregnancy after drug treatment.

## Acknowledgments

We are grateful to Dr Hu for her guidance on the manuscript.

## Author contributions

**Writing—original draft:** Congcong Qin.

**Writing—review & editing:** Zhijuan Hu.

**Supervision:** Yanan Shi, Hui Cui, Jiejie Li.
